# *Sasa veitchii* Extract Mitigates Mycophenolate Mofetil-Induced Human Palatal Cell Proliferation Inhibition by Downregulating *microRNA-4680-3p*

**DOI:** 10.3390/plants14071150

**Published:** 2025-04-07

**Authors:** Hanane Horita, Yosuke Tsukiboshi, Kenichi Ogata, Aya Ogata, Hisaka Kurita, Shuji Yamashita, Hirotaka Yamashita, Naoki Inagaki, Hyogo Horiguchi, Hiroki Yoshioka

**Affiliations:** 1 Faculty of Pharmacy, Gifu University of Medical Science, 4-3-3 Nijigaoka, Kani 509-0293, Gifu, Japan; 2 Section of Oral and Maxillofacial Oncology, Division of Maxillofacial Diagnostic and Surgical Sciences, Faculty of Dental Science, Kyushu University, 3-1-1 Maidashi, Higashi-ku, Fukuoka 812-8582, Fukuoka, Japan; 3 Laboratory of Medical Therapeutics and Molecular Therapeutics, Gifu Pharmaceutical University, 1-25-4 Daigakunishi, Gifu 501-1196, Gifu, Japan; 4 Laboratory of Community Pharmaceutical Practice and Science, Gifu Pharmaceutical University, 1-25-4 Daigakunishi, Gifu 501-1196, Gifu, Japan; 5 Department of Pharmacology, Graduate School of Medicine, University of the Ryukyus, 207 Uehara, Nishihara 903-0215, Okinawa, Japan; 6 Department of Hygiene, Kitasato University School of Medicine, 1-15-1 Kitasato, Minami-ku, Sagamihara 252-0374, Kanagawa, Japan

**Keywords:** cleft palate, *Sasa veitchii*, mycophenolate mofetil, microRNA, cell cycle

## Abstract

Cleft palate is a common birth defect worldwide and is caused by both genetic and environmental factors. Intrauterine drug exposure is one of the environmental factors that can induce cleft palate. Mycophenolate mofetil (MPM) is an immunosuppressant drug with teratogenic effects, including cleft palate. However, the research on MPM-induced cleft palate remains limited. *Sasa veitchii* extract (SE), a medical plant extract, is commercially available in Asia and has been reported to show effectiveness against oral diseases. The purpose of the present study is to evaluate whether SE protects against MPM-induced immunosuppression in human embryonic palatal mesenchymal (HEPM) cells. Cell viability and G1 phase-related cell cycle markers were assessed by co-treatment with MPM and SE. Furthermore, we quantified cleft palate-associated miRNA levels and the expression of its downstream genes. MPM treatment reduced cell viability in a concentration-dependent manner. Co-treatment with SE alleviated MPM-induced inhibition of HEPM cell proliferation. Additionally, SE reduced MPM-induced *miR-4680-3p* upregulation and the downregulation of its downstream genes (*ERBB2* and *JADE1*). These results suggest that SE alleviated MPM-induced cell proliferation inhibition through modulating *miR-4680-3p* expression.

## 1. Introduction

The early stage of pregnancy is a critical period for organogenesis in embryos. Exposure to teratogenic substances during this stage is considered a leading cause of severe congenital anomalies, such as cleft palate (CP) and microphthalmia [1,2]. Cleft lip (CL) with or without CP (CL/P) is a significant congenital defect, posing both functional and aesthetic challenges for children. It is recognized as the most common congenital anomaly globally, with an estimated incidence of approximately 1 in 700 live births [3]. The etiology of CL/P is complex, involving both environmental factors and genetic factors [4]. Given that surgical intervention is frequently the primary treatment for CL/P, it necessitates long-term care and substantial treatment costs. Consequently, preventive strategies, such as avoidance of teratogenic substances and folic acid supplementation, are recommended. Folic acid supplementation in early gestation has been shown to reduce the risk of neural tube defects [5] and has also been reported to decrease the incidence of CL/P, potentially by regulating transforming growth factor (TGF) β3 and mitigating oxidative stress [5,6,7].

Regarding genetic factors, several signaling pathways have been implicated in palate development [8,9]. The Wingless/Integrase-1 (WNT) signaling pathway is crucial for secondary palate formation, notably through *Paired box gene* (*Pax*) 9 regulation [10,11]. Mutations in *axis inhibition protein 2* have been linked to CL/P, as this protein regulates WNT signaling [12,13]. TGFβ3 promotes epithelial cell degradation via epithelial–mesenchymal transition (EMT) by inducing Snail1/2, a key transcription factor in EMT [14]. Studies have shown that Snail1/2-deficient mice exhibit reduced apoptosis in epithelial cells, leading to impaired palatal fusion [14]. The bone morphogenic protein signaling pathway is also essential for craniofacial morphogenesis, regulating critical cellular processes such as cell proliferation, differentiation, and apoptosis [15,16]. Recent literature reports that 131 human genes and 252 mouse genes have been associated with CP [17]. Concerning environmental factors, various maternal environmental exposures have been correlated with an elevated risk of CL/P [18]. These include occupational exposure to metals and pesticides [19], infections [20], smoking [21], and medication use during pregnancy [22]. These environmental factors can induce CL/P by disrupting essential genes or signaling pathways. For instance, maternal smoking has been associated with disruptions in TGF-β signaling, specifically through reduced TGF-α expression, thereby increasing the risk of CL/P [23,24].

In recent years, microRNAs (miRNAs)—small RNA molecules that regulate gene expression—have attracted significant attention. The first miRNA was discovered in 1993 [25,26], marking the inception of a new era in RNA biology. These ubiquitous molecules are present across diverse organisms, and to date, over 2500 miRNAs have been identified in the human genome [27]. Recent studies have highlighted the involvement of miRNAs in the epigenetic regulation of CL/P [28]. For instance, the *miR-17-92* clusters have been shown to control palatal mesenchyme cell proliferation and cell cycle [29]. Mutation of *miR-17-92* induces severe craniofacial abnormalities [30]. The *miR-146a* rs2910164 G allele has been found to regulate tumor necrosis factor receptor-associated factor 6 (*TRAF6*) expression, thereby contributing to the pathogenesis of CP [31]. Suzuki et al. reported that overexpression of *miR-374a-5p*, *miR-4680-3p*, and *miR-133b* suppresses human embryonic palatal mesenchymal (HEPM) cell through downregulation of CP-related genes [32]. Fu et al. demonstrated associations between *let-7c-5p*-*PIGA* and *miR-193a-3p*-*TGFB2* signaling pathways and HEPM cell viability [33].

Mycophenolate mofetil (MPM) is an immunosuppressant that selectively inhibits inosine monophosphate dehydrogenase [34]. Compared to conventional immunosuppressants such as azathioprine, MPM is associated with a lower incidence of adverse effects [35]. However, MPM has been reported to induce teratogenic effects, including CL/P and microtia [36]. Lin et al. reported that several proteins, including ribosomal protein 5, mouse double minute protein 2, and tumor suppressor p53, have been associated with MPM-induced CP [37]. We recently demonstrated that MPM reduced cell viability through the upregulation of *miR-4680-3p* and *let-7-5p* and the downregulation of their downstream genes in HEPM cells [38].

*Sasa veitchii* (Carrière) Rehder is a member of the Poaceae family and its extract preparation is commercially available as an over-the-counter drug, which is for recovering from fatigue, mouth sores, alveolar pyorrhea (periodontal disease), bad breath, and body odor in Japan [39]. In Asia, it has a history of traditional use in herbal medicines and dietary supplements, valued for its health-promoting properties [40]. Furthermore, *S. veitchii* extract (SE) has been shown to possess anti-inflammatory [40,41,42], anti-cancer [40,43], and anti-oxidant effects [40,41,44]. Notably, SE has been demonstrated to reduce the risk of periodontal disease and gingivitis [40,45]. Our previous research indicated that SE alleviated *all-trans*-retinoic acid (*at*RA)-induced cell proliferation inhibition through modulation of *miR-4680-3p* in HEPM cells [46]. Similarly, co-treatment with SE improved phenobarbital-induced cell viability reduction through the upregulation of TGF-β1 in human lip fibroblast cells [47]. Taken together, these findings suggest that SE may also exert protective effects against MPM-induced cell proliferation inhibition.

In this study, we aimed to investigate whether SE could alleviate MPM-induced cell proliferation inhibition using HEPM cells. We used commercial SE provided by Sunchlon Co. Ltd. (Nagano, Japan; Sunchlon^®^).

## 2. Results

### 2.1. MPM Inhibited HEPM Cell Proliferation in a Dose- and Time-Dependent Manner

**Figure 1:**
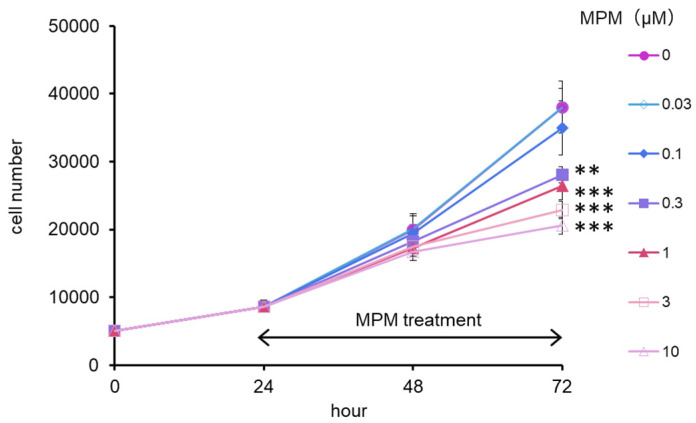
Proliferation of HEPM cells treated with MPM (0.03–10 µM) for 48 h. ** *p* < 0.01, and *** *p* < 0.001 versus control (n = 6).

Firstly, we evaluated the suppressive effect of MPM by treating it for 24 and 48 h on HEPM cells. As shown in Figure 1, the number of cells was reduced in a dose- and time-dependent manner and was significantly reduced in the MPM dose of 0.03–30 µM. For the following experiments, we selected 1 and 10 µM MPM for 48 h treatment.

### 2.2. SE Alleviated MPM-Induced Proliferation Inhibition in HEPM Cells

**Figure 2:**
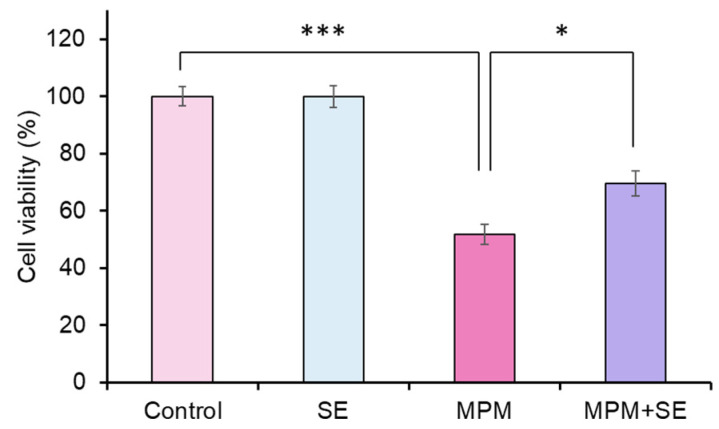
Protective effect of SE against MPM-induced inhibition of HEPM cell proliferation. 1 µM MPM and 100 µg/mL SE were used. * *p* < 0.05, and *** *p* < 0.001 versus control (n = 6).

We examined the protective effects of SE against MPM-induced cell proliferation reduction in HEPM cells. We found that treatment with SE (25, 50, 100 µg/mL) did not affect HEPM cell viability (Supplementary Figure S2). While treatment with 1 and 10 µM MPM reduced cell viability (Supplementary Figure S2), co-treatment with SE alleviated MPM-induced cell proliferation inhibition in a dose-dependent manner (Supplementary Figure S2). Of note, we demonstrated that co-treatment with 100 µg/mL SE significantly alleviated MPM-induced cell viability reduction (*p* < 0.05, Figure 2).

### 2.3. Co-Treatment with Sodium Copper Chlorophyllin (SCC) Failed to Recover MPM-Induced Cell Proliferation Reduction in HEPM Cells

**Figure 3:**
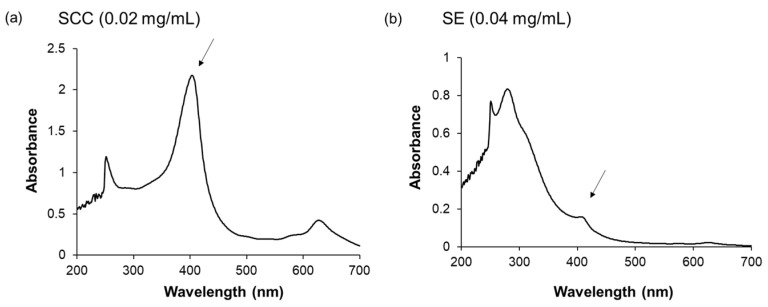
The absorption spectra of SCC and SE. (**a**) indicated SCC (0.02 mg/mL) and (**b**) indicated SE (0.04 mg/mL). The arrow shows the main peak of SCC.

We further focused on the protective effects of SCC against MPM-induced cell proliferation inhibition in HEPM cells since the main component of SE is SCC (0.25%) [39]. We tested the ingredients of SE using infrared absorption spectrometry analysis and found that a SCC peak at around 400 nm was seen in both samples (Figure 3). We also demonstrated that broad peak around 250–300 nm was detected in SE (Figure 3b). These data suggest that the SE we used contained many compounds.

**Figure 4:**
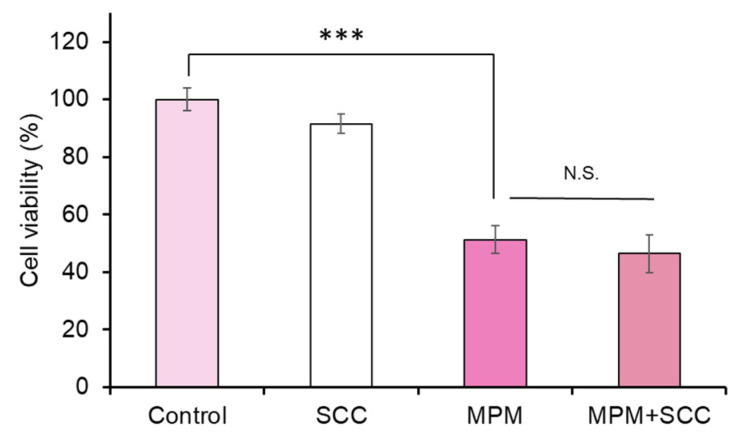
Sodium copper chlorophyllin failed to alleviate MPM-induced cell proliferation inhibition in HEPM cells. 1 µM MPM and 1 µg/mL SCC were used. *** *p* < 0.001 (n = 6). N.S.; Not significant.

We found that treatment with SCC (0.3 and 1 µg/mL [1.2 and 4 times compared to 100 μg/mL SE]) did not change the HEPM cell number. Co-treatment with SCC failed to recover against MPM-induced cell proliferation inhibition in HEPM cells (Figure 4). This result suggests that ingredients other than SCC contribute to the protective effect of SE.

### 2.4. SE Alleviated MPM-Induced Cell Cycle Arrest in HEPM Cells

**Figure 5:**
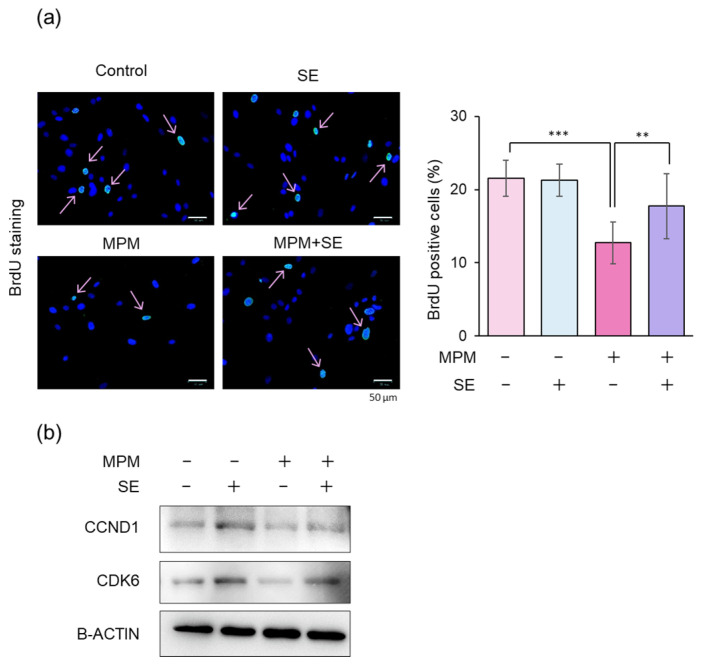
SE attenuated MPM-induced cell cycle arrest in HEPM cells. (**a**) BrdU staining (green) of HEPM cells after treatment with 1 µM MPM and/or 100 µg/mL SE for 48 h. BrdU-positive cells were stained green, and nuclei were counterstained with 4′,6-diamidino-2-phenylindole (blue). Arrow indicated BrdU-positive cells. Scale bar, 50 µm. The graph shows the quantification of BrdU-positive cells. ** *p* < 0.01 and *** *p* < 0.001 (n = 8–10). (**b**) Western blotting of HEPM cells treated with 1 µM MPM and/or 100 µg/mL SE for 48 h. β-ACTIN served as an internal control.

We tested the BrdU incorporation assay since we previously demonstrated that MPM-induced cell number reduction was G1 cell cycle arrest, not apoptosis-induced cell death [38]. We found that BrdU-positive cells significantly decreased by treatment with 1 µM MPM, while co-treatment with SE (100 µg/mL) significantly rescued the MPM-induced inhibition of BrdU incorporation (Figure 5a). To further investigate the molecular mechanism of MPM-induced cell cycle arrest (G1-arrest), we tested cyclins and cyclin-dependent kinases (CDK) by immunoblotting (Figure 5b). We found that MPM treatment reduced CCND1 and CDK6 levels. Moreover, treatment with SE induced these protein levels. These results suggest that SE alleviated MPM-induced cell cycle arrest associated with CCND1/CDK6 upregulation in HEPM cells.

### 2.5. SE Downregulated miR-4680-3p and Upregulated Its Downstream Genes in HEPM Cells

**Figure 6:**
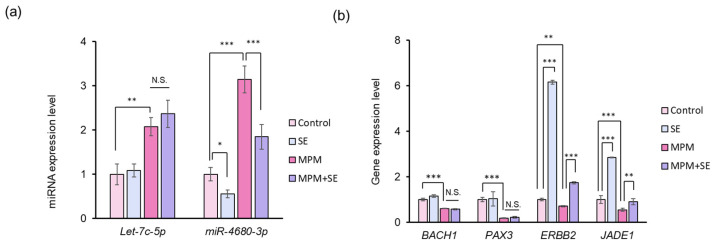
SE downregulated of *miR-4680-3p* levels and upregulated of *ERBB2* and *JADE1* in HEPM cells. (**a**) Quantitative RT-PCR analysis of *Let-7c-5p* and *miR-4680-3p* expression after treatment with 1 µM MPM and/or 100 µg/mL SE in HEPM cells. * *p* < 0.05, ** *p* < 0.01, and *** *p* < 0.001. N.S.; Not Significant. (**b**) Quantitative RT-PCR analysis of *BACH1*, *PAX3*, *ERBB2*, and *JADE1* expression after treatment with 1 µM MPM and/or 100 µg/mL SE in HEPM cells. ** *p* < 0.01, and *** *p* < 0.001. N.S.; Not Significant.

**Figure 7:**
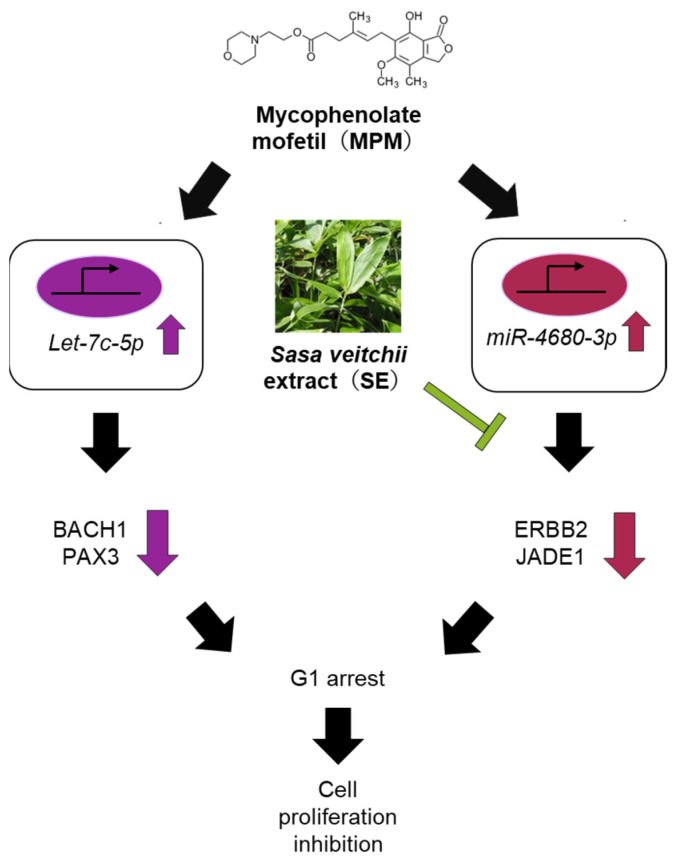
Proposed mechanism of SE against MPM-induced cell proliferation inhibition.

Finally, we investigated the miRNA expression level by treatment with SE since we recently reported that MPM-induced inhibition of HEPM cell proliferation occurs through upregulation of *let-7c-5p* and *miR-4680-3p* expression [38]. We found that the upregulation of *let-7c-5p* and *miR-4680-3p* expression was seen by MPM (Figure 6a). Additionally, we revealed that SE significantly downregulated the expression of *miR-4680-3p* in HEPM cells. Co-treatment with SE significantly alleviated *miR-4680-3p* expression levels in HEPM cells. In contrast, *let-7c-5p* expression level was not altered by treatment with SE (Figure 6a). To further investigate the effects of *miR-4680-3p* and *let-7c-5p*, we conducted a quantitative RT-PCR analysis. We found that MPM treatment significantly suppressed *BACH1*, *PAX3, ERBB2*, and *JADE1* expression levels (Figure 6b). SE treatment significantly upregulated *ERBB2* and *JADE1* expression levels, while *BACH1* and *PAX3* expression levels were not changed. Moreover, co-treatment with SE significantly increased the *ERBB2* and *JADE1* expression levels compared to MPM treatment. These results indicated that SE exerts the protective effect via modulation of *miR-4680-3p*-*ERBB2*/*JADE1* expression (Figure 7).

## 3. Discussion

MPM is a type of immunosuppressant that prevents cell proliferation and autoimmunity. MPM induces G1 cell cycle arrest and results in a loss of the G_2_/M phase peak, leading to growth inhibition in osteosarcoma U2Os cells [48]. MPM decreases the mesangial cell numbers through the downregulation of CCND1 [49]. MPM induces G1-S phase cell cycle arrest in multiple myeloma cells [50]. We previously reported that MPM reduced human lip fibroblast cell viability associated with CCND1/CDK6 [51]. In addition, we recently showed that cyclin and cyclin-dependent kinase was downregulated by MPM in HEPM cells [38]. In the present study, we demonstrated that MPM-induced CCND1 and CDK6 reduction was recovered by co-treatment with SE. These findings suggest that SE alleviated MPM-induced cell viability reduction through the regulation of CCND1 and CDK6.

Recent reports suggest that miRNA is associated with CL/P [9,52]. Suzuki et al. and Li et al. showed that miRNA was predicted using CP-related genes and bioinformatics analysis. They found that the overexpression of *miR-133b*, *miR-140-5p*, *miR-374-5p*, *miR-381a-3p*, and *miR-4680-3p* suppresses cell proliferation in HEPM cells by regulating target genes and signaling pathways [17,32]. Fu et al. reported that *let-7c-5p* and *miR-193a-3p* were identified in the database of CP patients and overexpression of *let-7c-5p* and *miR-193a-3p* reduced HEPM cell viability [33]. As an environmental factor, a relationship between medication intake-induced CP and miRNA was reported. Zhou et al. reported that *at*RA treatment upregulated *miR-470-5p* expression and suppressed EMT of mouse embryonic palatal shelf epithelial cells [53]. Zhang et al. showed that upregulation of *miR-106a-5p* by *at*RA induced apoptosis through regulation of the TGFb/Smad signaling pathway in mice [54]. Inhibition of *miR-4680-3p* restored *at*RA-induced HEPM cell viability reduction [55]. *miR-130a-3p* significantly contributes to the inhibition of mouse embryonic palatal mesenchymal cell proliferation induced by dexamethasone [56]. *miR-4680-3p* induction was associated with phenytoin-induced inhibition of cell proliferation in HEPM cells [57]. We recently found that *let-7c-5p* and *miR-4680-3p* were upregulated among the above seven miRNAs in HEPM cells and inhibition of *let-7c-5p* and *miR-4680-3p* alleviated MPM-induced cell proliferation inhibition [38]. In the present study, we confirmed the upregulation of *let-7c-5p* and *miR-4680-3p* by treatment with MPM. Among the two miRNAs, we found that *miR-4680-3p* was significantly reduced by co-treatment with SE, while *let-7c-5p* expression levels were not changed. These results indicated that the SE-induced protective effect was through the modulation of *miR-4680-3p*. Since we previously reported that *at*RA-induced cell proliferation inhibition was attenuated by co-treatment with SE through modulation of *miR-4680-3p* [45], the present mechanism is reasonable.

The reports related to the *miR-4680-3p* function were limited compared to *let-7c-5p* [58,59]. As far as we know, Suzuki et al. first demonstrated that overexpression of *miR-4680-3p* reduced cell viability in HRPM cells [32] and the same research group found that *at*RA-induced *miR-4680-3p* upregulation was associated with HEPM cell proliferation through modulation of downstream genes (*ERBB2* and *JADE1*) [55] (Supplementary Figure S2). ERBB2 is a part of the ERBB receptor tyrosine kinase family, which also includes the epidermal growth factor receptor [60]. When ligands bind to these receptors, it induces the homo- or heterodimerization, activating the kinase domain. This activation initiates downstream signaling cascades, such as mitogen-activated protein kinase/extracellular signal-regulated kinase and phosphatidylinositol-3 kinase/protein kinase B/mechanism of rapamycin pathways, both of which are crucial for cell proliferation, migration, and differentiation [61,62]. The overexpression of *ERBB2* leads to a reduction in the G1 phase of the cell cycle by promoting the levels of CDK6, CCND1, and CCNE [63]. As for the palatal shelf, the bioinformatic analysis suggested that the ERBB signaling pathway may play a significant role in the formation of the palate [64]. In addition, we recently demonstrated that MPM inhibits cell proliferation of HEPM cells by upregulating *miR-4680-3p* expression, and downregulating ERBB2 expression-induced G1 phase arrest [38]. *JADE1*, also known as PHF17, is a transcription factor and contains two variants: JADE1-L, which is a long form with 842 amino acids, and JADE1-S, which is a short form without a C-terminal fragment of 333 amino acids [65,66]. The knockdown of *JADE1* (both variants) by siRNA results in inhibition of DNA synthesis in human non-small cell lung carcinoma cell line (h1299 cells) and primary fibroblasts [67]. Although the role of *JADE1* remains elusive, the protein exhibits histone acetyltransferase (HAT) activity and acts as a co-factor of the HBO1 complex in histone H4 acetylation during gene regulation, which is essential for regulating the cell cycle [68,69]. *JADE1* regulates the WNT/β-catenin signaling pathway [70,71]. Since CCND1 is a downstream gene in this pathway [72], *JADE1* may indirectly control CCND1. In the present study, we found that co-treatment with SE recovered MPM-induced ERBB2 and JADE1 expression reduction and CCND1 and CDK6 downregulation. These results suggest that SE-induced *ERBB2* and *JADE1* upregulation plays a crucial role in cell proliferation inhibition against MPM in HEPM cells.

According to the company data, SCC (0.25 %) is the main component of SE [39]. SCC has various potential effects, including antimutagenic [73], anti-carcinogenic [74], and antioxidant activities [75]. The total antioxidant status in patients with CL/P was lower than those in the control group (healthy people) [76], and CL/P may be related to oxidation stress [77]. Therefore, we hypothesized that the protective effect of SE is due to the presence of SCC. However, our results failed to alleviate the toxic effect of MPM by co-treatment with SCC in HEPM cells. This result indicated that the protective effect of SE was due to the presence of ingredients other than SCC. This conclusion is corroborated by the results of our previous study since we analyzed SE by 3D-high-performance liquid chromatography and showed the inclusion of several peaks other than SCC in the chromatogram [47]. *S*. species include various phenolic compounds such as flavonoids, including myricetin, vitexin and luteolin, and phenolic acids such as coumaric acid, which have antioxidant capacity [40,78,79]. Moreover, several reports have shown that various SE compounds involve miRNA expression. Chung et al. found that tricin suppresses cell proliferation by increasing *miR-7* in C6 glioma cells [80]. Myricetin attenuated hepatic steatosis by regulating *miR-146b* [81] and inflammatory response by inducing *miR-29a-3p* [82]. Vitexin-induced apoptosis and oxidative stress were associated with specific miRNAs such as the let-7c family, *miR-17-5p*, and *miR-495* [83,84]. Coumaric acid has antitumor and anti-inflammatory effects by regulating *miR-7-5p, miR-30a-5p, miR-125a-5p, and miR-146a* [85,86]. Luteolin has antitumor effects through *miR-34a-5p* regulation [87]. Although further investigation is needed, we concluded that compounds in the SE, such as flavonoids, and coumaric acid, exert protective effects through miRNA or oxidative regulation. In the future, we need to measure the content of phenolic compounds from SE and identify the active compounds of SE using LC-MS/MS.

While this study provides valuable insights, it is important to acknowledge two limitations. Firstly, the active components within SE are yet to be fully elucidated. Further research will focus on identifying these components, initially through fractionation and subsequent bioactivity-guided assays. Secondly, the study is limited to in vitro experiments. In vivo studies are crucial to comprehensively evaluate the protective efficacy of SE in a living system. Although SE is approved as an over-the-counter medicine in Japan, we need to evaluate the toxic effect of SE during the pregnancy period. Notwithstanding these limitations, the present investigation provides a valuable initial assessment of SE’s protective effects against MPM-induced inhibition of cell proliferation in HEPM cells.

## 4. Materials and Methods

### 4.1. Cell Culture

HEPM cells were purchased from the JCRB Cell Bank (JCRB9095, Osaka, Japan) and maintained in Minimum Essential Medium Eagle-alpha modification (αMEM; Fujifilm-Wako Pure Chemical Corporation, Osaka, Japan) supplemented with 10% fetal bovine serum (Millipore-Sigma, St Louis, MO, USA), penicillin (10 U/mL), and streptomycin (10 μg/mL; Nacalai Tesque, Kyoto, Japan). The cells were maintained at 37 °C in a humidified atmosphere containing 5% CO_2_.

### 4.2. Preparation of SE

SE was kindly gifted from Sunchlon Co. Ltd. (Nagano, Japan). Fresh leaves of *S. veitchii* were cut into small pieces, and the magnesium in the chlorophyllin was replaced by copper by soaking the leaves in boiled water containing copper sulfate. This substitution prevents the decomposition of chlorophyllin. The cell walls were then hydrolyzed by boiling the leaves in a 15% (*w*/*v*) sodium hydroxide solution for 80 min. Afterward, hydrochloric acid was added to the resulting solution, which contained hydrolyzed cell walls and cytoplasmic components, causing a precipitate to form. The precipitate was collected by centrifugation and dissolved by adding sodium hydroxide until the pH reached 7. According to the manufacturer’s data, the SE solution is an extract derived from *S. veitchii* leaves, with 1 mL of the solution containing an equivalent of 2.82 g of the leaves. We freeze-dried the SE solution. We obtained 9.44 g of powdered SE from 120 mL of SE solution (Sunchlon^®^, lot# 55222) [43].

### 4.3. Infrared Absorption Spectrometry Analysis

The absorption spectra were measured using a Shimadzu UV-2600i UV-Vis spectrophotometer (Shimazu, Tokyo, Japan). The sample concentrations were SE (0.04 mg/mL) and SCC (0.02 mg/mL).

### 4.4. Cell Proliferation Assay

HEPM cells were plated in 96-well plates at a density of 5000 cells/well (n = 6) and treated with various concentrations (0–10 μM) of MPM (Tokyo Kasei Co. Ltd., Tokyo, Japan) after 24 h of cell seeding. After treatment with MPM for 24, or 48 h, the cell viability was evaluated using Alamar Blue (Bio-Rad Laboratories, Hercules, CA, USA). For the rescue experiment, HEPM cells were plated in 96-well plates at a density of 5000 cells/well (n = 6) and treated with 0, 1, or 10 μM MPM and 25, 50, or 100 μg/mL SE or 0.3, or 1 μg/mL SCC (Nacalai Tesque) after 24 h of cell seeding. After 48h of treatment, cell viability was measured in the presence or absence of SE.

### 4.5. Bromodeoxyuridine (BrdU) Incorporation Assay

HEPM cells were plated on 8-well chamber slides (Biomedical Sciences Inc., Tokyo, Japan) at a density of 10,000 cells/well and treated with 1 μM MPM, 100 μg/mL SE, 1 μM MPM + 100 μg/mL SE, or vehicle. After 48 h of treatment, the cells were incubated with BrdU (100 μg/mL) for 40 min. The incorporated BrdU was stained with an anti-BrdU antibody (1:150, Santacruz Biotechnology, Dallas, TX, USA) and fluorescein (FITC)-conjugated anti-mouse IgG (1:180; MBL, Aichi, Japan). Nuclei were counterstained with 4′,6-diamidino-2-phenylindole (DAPI, Nacalai Tesque), and BrdU-positive cells were quantified in 6–8 fields.

### 4.6. Western Blot Analysis

HEPM cells were plated in a 35 mm dish at a density of 2 × 10^5^ cells per dish and treated with 1 µM MPM, 100 µg/mL SE, 1 µM MPM + 100 µg/mL SE, or vehicle after 24 h cell seeding. After 48 h of treatment, we washed the phosphate-buffered saline (PBS) twice and added 100 µL ice-cold RIPA buffer (Nacalai Tesque) containing a protease inhibitor cocktail (Nacalai Tesque) and waited 5 min on ice. It was subsequently scraped and centrifuged (20,000× *g* for 20 min at 4 °C) as previously described [38,56]. Protein samples (10 µg) were subjected to 10% sodium dodecyl sulfate-polyacrylamide gel electrophoresis and transferred onto polyvinylidene difluoride membranes. Anti-mouse cyclin D1 (CCND1) antibody (1:1000 dilution; Santa Cruz Biotechnology, Dallas, TX, USA), anti-mouse cyclin-dependent kinase 6 (CDK6) antibody (1:2000 dilution; Proteintech Japan, Tokyo, Japan) and anti-mouse b-actin monoclonal antibodies (1:3000 dilution; MBL, Aichi, Japan) were used as primary antibodies for immunoblotting. A peroxidase-conjugated anti-rabbit immunoglobulin G (IgG) and a peroxidase-conjugated anti-mouse IgG (Cell Signaling Technology) were used as secondary antibodies (1:10,000 dilution). The immunoreactive bands were visualized by Western Blot Hyper HRP Substrate (Takara Bio, Shiga, Japan).

### 4.7. Quantitative RT-PCR

HEPM cells were plated in 35 mm dish at a density of 2 × 10^5^ cells per dish and treated with 1 μM MPM and/or 100 μg/mL SE or vehicle after 24 h of cell seeding. After 48 h of treatment, we washed PBS twice, and total RNA was extracted using a QIAshredder and miRNeasy Mini Kit (QIAGEN, Valencia, CA, USA) as we previously described [57,88]. Total RNA (25 ng) was reverse transcribed using a miRNA Reverse Transcription Reaction Kit (GeneCopoeia, Rockville, MD, USA). miRNA expression was examined using an all-in-one miRNA qRT-PCR Detection Kit (GeneCopoeia). Probe information and PCR conditions were as previously described [38,57].

### 4.8. Statistical Analyses

Comparisons between more than two groups were performed using Tukey’s test. Cell viability assay for multiple groups was evaluated using a two-way analysis of variance with Dunnett’s test. All statistical analyses were performed using SPSS Statistics for Windows (version 26.0; IBM Corp., Armonk, NY, USA). Differences were considered statistically significant at *p* < 0.05.

## Data Availability

All relevant data are within the manuscript.

## Supplementary Materials

The following supporting information can be downloaded at: https://www.mdpi.com/article/10.3390/plants14071150/s1, Figure S1: Effect of SE and SCC against MPM-induced cell proliferation inhibition in HEPM cells; Figure S2: Putative target site for *miR-4680-3p* in the ERBB2 and JADE1 3′ UTR.

## Abbreviations

The following abbreviations are used in this manuscript:

*at*RA	*all-trans*-retinoic acid
CDK	cyclin-dependent kinases
CL	Cleft lip
CP	Cleft palate
CL/P	Cleft lip with or without cleft palate
EMT	epithelial-mesenchymal transition
HEPM	human embryonic palatal mesenchymal
HPLC	High performance liquid chromatography
miRNA	microRNA
MPM	Mycophenolate mofetil
Pax	Paired box gene
SE	*Sasa veitchii* extract
SCC	sodium copper chlorophyllin
TGF	transforming growth factor
WNT	Wingless/integrase-1
