# Supplementary material for: Sasa veitchii Extract Mitigates Mycophenolate Mofetil-Induced Human Palatal Cell Proliferation Inhibition by Downregulating microRNA-4680-3p
Source: Plants (Basel). 2025 Apr 7;14(7):1150. doi: 10.3390/plants14071150 (PMC11991523; doi:10.3390/plants14071150)
Supplement: Supplementary file 1 [file plants-14-01150-s001.zip › plants-3527467-supplementary.pdf]

Supplementary Figure S1

```
ERBB2 726-732        5' ...GAACAAAAGCGACCCAUUCAGAG...
                          | | | | |            | | | | | |
hsa-miR-4680-3p      3'   AUUGUUGAGAAUGU-UAAGUCU

JADE1 121-128        5' ...AACACAUUUACUUGC--AAUUCAGA...
                                  | | |       | | | | | | |
hsa-miR-4680-3p      3'      AUUGUUGAGAAUGUUAAGUCU
```

Supplementary Figure S2

(a)

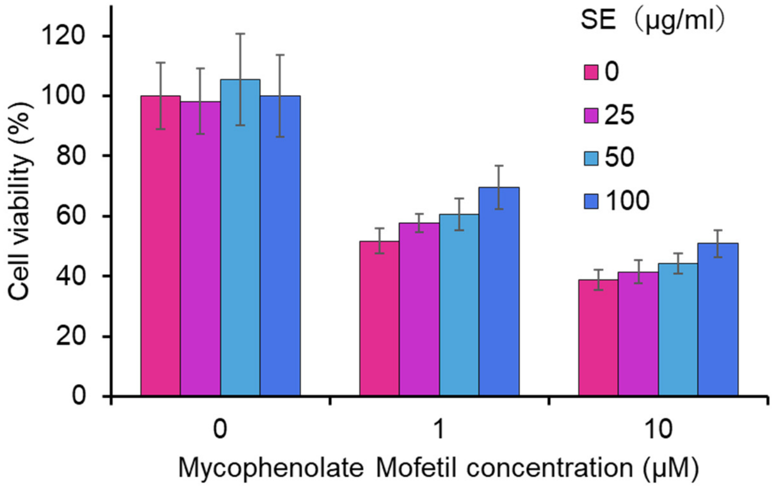

(b)

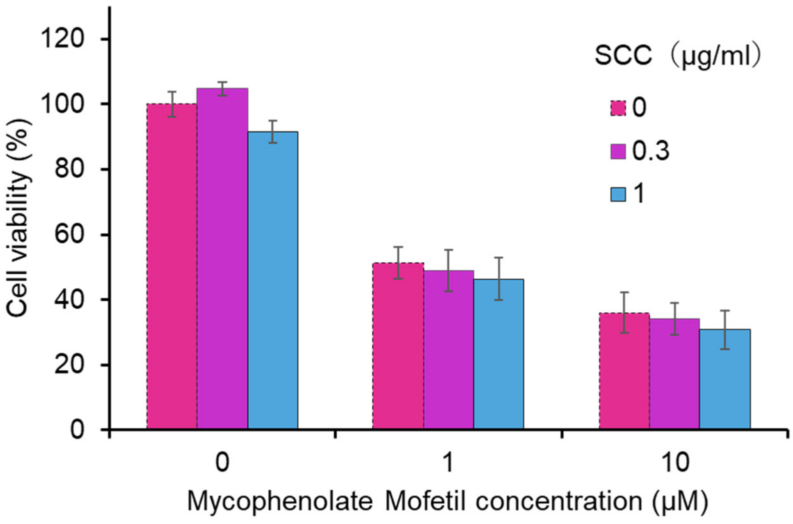

Supplementary Figure Legends

Supplemental Figure S1. Putative target site for *miR-4680-3p* in the ERBB2 and JADE1 3' UTR.

TargetScan (Version 8.0) was used for the prediction of target genes

Supplemental Figure S2. Effect of SE and SCC against MPM-induced cell proliferation inhibition in HEPM cells.

(a) 1 and 10 μM MPM and 25, 50, and 100 μg/mL SE was used for the experiment.
(b) 1 and 10 μM MPM and 0.3, and 1 μg/mL SCC was used for the experiment.
